# Dimethyl dl-2,3-dibenzyl-2,3-diisothio­cyanatosuccinate

**DOI:** 10.1107/S1600536812009294

**Published:** 2012-03-10

**Authors:** Justyna Kalinowska-Tłuścik, Dariusz Cież, Sandrine Peyrat

**Affiliations:** a Faculty of Chemistry, Jagiellonian University, R. Ingardena 3, 30-060, Kraków, Poland

## Abstract

The title compound, C_22_H_20_N_2_O_4_S_2_, has approximate mol­ecular twofold symmetry. In the crystal, the presence of C—H⋯π inter­actions leads to the formation of *zigzag* chains along [001].

## Related literature

For the synthesis and spectroscopic characterization of the title compound, see: Cież (2007 ▶). For the synthesis, spectroscopic characterization and crystal structure determination of similar compounds, see: Cież *et al.* (2008 ▶). For diisothio­cyanates, see: Morel & Marchand (2001 ▶). For C—H⋯ π and C—H⋯O inter­actions, see: Malone *et al.* (1997 ▶); Arunan *et al.* (2011*a* ▶,*b* ▶).
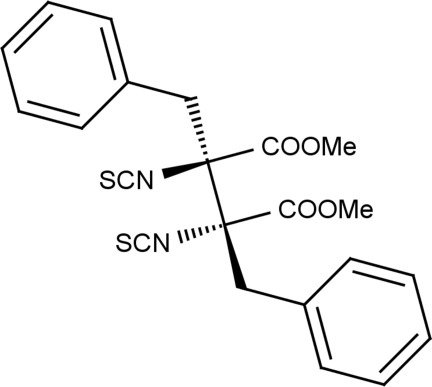


## Experimental

### 

#### Crystal data

C_22_H_20_N_2_O_4_S_2_
*M* *_r_* = 440.52
Monoclinic, 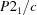

*a* = 9.1658 (1) Å
*b* = 19.3999 (4) Å
*c* = 12.2762 (2) Å
β = 97.891 (1)°
*V* = 2162.23 (6) Å^3^
*Z* = 4
Mo *K*α radiation
μ = 0.28 mm^−1^
*T* = 100 K
0.28 × 0.18 × 0.18 mm

#### Data collection

Nonius KappaCCD diffractometer
Absorption correction: multi-scan (*DENZO-SMN*; Otwinowski & Minor, 1997 ▶) *T*_min_ = 0.926, *T*_max_ = 0.952
9175 measured reflections
4868 independent reflections
4124 reflections with *I* > 2σ(*I*)
*R*_int_ = 0.021

#### Refinement

*R*[*F* ^2^ > 2σ(*F* ^2^)] = 0.037
*wR*(*F* ^2^) = 0.090
*S* = 1.05
4868 reflections
273 parameters
H-atom parameters constrained
Δρ_max_ = 0.35 e Å^−3^
Δρ_min_ = −0.52 e Å^−3^

### 

Data collection: *COLLECT* (Nonius, 1998 ▶); cell refinement: *HKL* *SCALEPACK* (Otwinowski & Minor, 1997 ▶); data reduction: *HKL* *DENZO* (Otwinowski & Minor, 1997 ▶) and *SCALEPACK*; program(s) used to solve structure: *SIR92* (Altomare *et al.*, 1994 ▶); program(s) used to refine structure: *SHELXL97* (Sheldrick, 2008 ▶); molecular graphics: *ORTEP-3 for Windows* (Farrugia, 1997 ▶); software used to prepare material for publication: *WinGX* (Farrugia, 1999 ▶), *MarvinSketch* (Chemaxon, 2010 ▶) and *publCIF* (Westrip, 2010 ▶).

## Supplementary Material

Crystal structure: contains datablock(s) global, I. DOI: 10.1107/S1600536812009294/fj2516sup1.cif

Structure factors: contains datablock(s) I. DOI: 10.1107/S1600536812009294/fj2516Isup2.hkl

Supplementary material file. DOI: 10.1107/S1600536812009294/fj2516Isup3.mol

Supplementary material file. DOI: 10.1107/S1600536812009294/fj2516Isup4.cml

Additional supplementary materials:  crystallographic information; 3D view; checkCIF report

## Figures and Tables

**Table 1:** Hydrogen-bond geometry (Å, °) *Cg* is the centroid of the C32–C37 ring.

*D*—H⋯*A*	*D*—H	H⋯*A*	*D*⋯*A*	*D*—H⋯*A*
C38—H21*C*⋯*Cg*^i^	0.98	2.61	3.461 (2)	145

Symmetry code: (i) 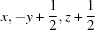.

**Fig. 1.**
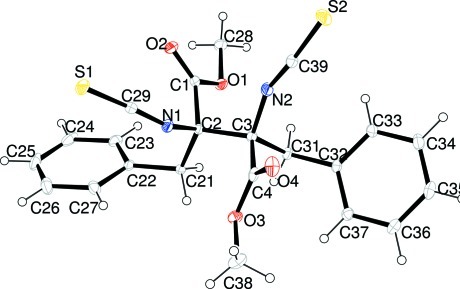
Asymmetric unit of the title compound - here R,R - enantiomer, showing the molecule conformation. Atom displacement ellipsoids drawn at the 30% probability level and H atoms are shown as spheres of arbitrary radii.

**Fig. 2.**
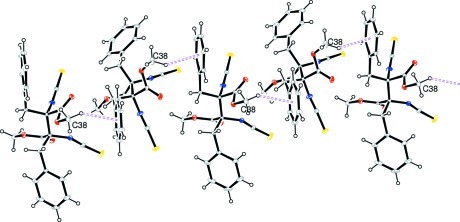
Chain formed by C—H···π interacting molecules, propagating in the [001] direction.

## supplementary crystallographic information

### Comment

The title compound was synthesized as a part of a larger project focusing on the synthesis of 2,3-disubstituted 2,3-diaminosuccinic acid derivatives obtained from titanium (IV) enolates of 2-isothiocyanato-carboxylates *via* C—C bond formation in oxidative homo-coupling of titanium (IV) enolates of 2-isothiocyanato-carboxylic esters (Cież, 2007; Cież *et al.*, 2008). The main reason for the interest in vicinal diisothiocyanates is related to their wide application in organic syntheses (Morel & Marchand, 2001). The molecule, crystal structure of which is presented here, belongs to the rare class of organic compounds.

The overall shape of the title molecule is shown in Figure 1. There is pseudo-symmetry in the molecule (2-fold axis perpendicular to C2—C3 bond and parallel to [212]). The mutual orientation of both isothiocyanate groups, same as both benzyl groups, is *gauche* with dihedral angels N1—C2—C3—N2 = 73.95 (13)° and C21—C2—C3—C31 = 43.41 (16)°. The ester groups are oriented in *anti* conformation with dihedral angle C1—C2—C3—C4 = 158.31 (11)°.

There are two chiral centres in the molecule, localized on atoms C2 and C3, both with the same absolute configuration (*R*,*R* enantiomer shown in Figure1).

The crystal structure of the title compound is stabilized by weak interactions. The strongest are C—H···π interactions (Arunan *et al.*, 2011*a*; Arunan *et al.*, 2011*b*; Malone *et al.*, 1997). They are formed between molecules related *via* glide plane c. The distance between hydrogen atom and centroid of the aromatic ring (Cg) is 2.611Å, with angle C38—H···Cg = 145.17°. The additionally defined angle of approach of the vector HCg to the plane of the aromatic ring, θ = 77°, and horizontal distance 0.6Å, classify this C—H···π as the second common geometry for this type of interaction observed in crystal structures (type III according to Malone *et al.*, 1997). Intermolecular C—H···π interactions between neighbouring molecules observed in this structure form a *zigzag-*like chain in the [001] direction (Figure 2), where only one aromatic ring of the title molecule is involved.

Additional interaction is observed between C31—H···O4^i^ [where (i) is *x*, -*y* + 1/2, *z* + 1/2] with C···O = 2.985 (2)Å, H···O = 2.612Å and angle C—H···O = 102.35°. The parameter suggests that it is not a hydrogen bond (Arunan *et al.*, 2011*a*; Arunan *et al.*, 2011*b*), however this interaction plays a crucial role in the stabilization of the methyl group (C38), allowing for above mentioned C—H···π. What is interesting, the sulphur atoms of the thiocyanate groups are not involved in intermolecular interactions.

The second aromatic moiety of the DL-2,3-dibenzyl-2,3-diisothiocyanato-succinic acid dimethyl ester is not involved in C—H···π. It is placed in short distance to a corresponding ring of the neighbouring molecule, related *via* the inversion centre (C24···C24^ii^ = 3.390 (2)Å, where (ii) is -*x* + 2, -*y*, -*z* + 2). However, the overlapping of the aromatic rings is not observed. This suggests a hydrophobic association.

### Experimental

The title compound was obtained by oxidative homo-coupling of methyl (*S*)-2-isothiocyanato-3-phenyl-propionate in TiCl_4_/DIEA (*N*,*N*-diisopropylethylamine) system at 177 K and characterized by NMR spectroscopy (Cież, 2007). Colourless, block single crystals suitable for X-ray diffraction were obtained from ethanol solution by slow evaporation of solvent at ambient conditions.

### Refinement

All non-hydrogen atoms were refined anisotropically using weighted full-matrix least-squares on F^2^. All hydrogen atoms were calculated at idealized positions and refined using a riding model with C—H = 0.95Å and *U*_iso_(H) = 1.2*U*_eq_(C) for aromatic hydrogen atoms, C—H = 0.99Å and *U*_iso_(H) = 1.2*U*_eq_(C) for methylene groups, C—H = 0.98Å and *U*_iso_(H) = 1.5*U*_eq_(C) for the methyl groups refined as rotating group.

### Crystal data

**Table:**

C_22_H_20_N_2_O_4_S_2_	*F*(000) = 920
*M**_r_* = 440.52	*D*_x_ = 1.353 Mg m^−^^3^
Monoclinic, *P*2_1_/*c*	Melting point: 405(1) K
Hall symbol: -P 2ybc	Mo *K*α radiation, λ = 0.7107 Å
*a* = 9.1658 (1) Å	Cell parameters from 8876 reflections
*b* = 19.3999 (4) Å	θ = 1.0–27.5°
*c* = 12.2762 (2) Å	µ = 0.28 mm^−^^1^
β = 97.891 (1)°	*T* = 100 K
*V* = 2162.23 (6) Å^3^	Block, colourless
*Z* = 4	0.28 × 0.18 × 0.18 mm

### Data collection

**Table:**

Nonius KappaCCD diffractometer	4868 independent reflections
Radiation source: fine-focus sealed tube	4124 reflections with *I* > 2σ(*I*)
Graphite monochromator	*R*_int_ = 0.021
Detector resolution: 9 pixels mm^-1^	θ_max_ = 27.5°, θ_min_ = 2.7°
CCD scans	*h* = 0→11
Absorption correction: multi-scan (*DENZO-SMN*; Otwinowski & Minor, 1997)	*k* = −25→24
*T*_min_ = 0.926, *T*_max_ = 0.952	*l* = −15→15
9175 measured reflections	

### Refinement

**Table:**

Refinement on *F*^2^	0 restraints
Least-squares matrix: full	H-atom parameters constrained
*R*[*F*^2^ > 2σ(*F*^2^)] = 0.037	*w* = 1/[σ^2^(*F*_o_^2^) + (0.033*P*)^2^ + 1.1739*P*] where *P* = (*F*_o_^2^ + 2*F*_c_^2^)/3
*wR*(*F*^2^) = 0.090	(Δ/σ)_max_ = 0.001
*S* = 1.05	Δρ_max_ = 0.35 e Å^−^^3^
4868 reflections	Δρ_min_ = −0.52 e Å^−^^3^
273 parameters	

### Special details

**Table:**

Geometry. All s.u.'s (except the s.u. in the dihedral angle between two l.s. planes) are estimated using the full covariance matrix. The cell s.u.'s are taken into account individually in the estimation of s.u.'s in distances, angles and torsion angles; correlations between s.u.'s in cell parameters are only used when they are defined by crystal symmetry. An approximate (isotropic) treatment of cell s.u.'s is used for estimating s.u.'s involving l.s. planes.
Refinement. Refinement of *F*^2^ against ALL reflections. The weighted *R*-factor *wR* and goodness of fit *S* are based on *F*^2^, conventional *R*-factors *R* are based on *F*, with *F* set to zero for negative *F*^2^. The threshold expression of *F*^2^ > σ(*F*^2^) is used only for calculating *R*-factors(gt) *etc*. and is not relevant to the choice of reflections for refinement. *R*-factors based on *F*^2^ are statistically about twice as large as those based on *F*, and *R*- factors based on ALL data will be even larger.

### Fractional atomic coordinates and isotropic or equivalent isotropic displacement parameters (Å^2^)

**Table:**

	*x*	*y*	*z*	*U*_iso_*/*U*_eq_	
S1	0.82190 (5)	0.10092 (2)	0.64722 (4)	0.03234 (12)	
S2	0.70194 (5)	0.41656 (2)	0.81742 (4)	0.03349 (12)	
O1	0.77379 (11)	0.19649 (6)	1.07866 (8)	0.0215 (2)	
O3	0.32504 (11)	0.16901 (6)	0.82274 (8)	0.0220 (2)	
O2	0.89922 (11)	0.18316 (6)	0.93462 (9)	0.0259 (2)	
O4	0.37916 (13)	0.26642 (6)	0.73636 (9)	0.0296 (3)	
N1	0.65160 (13)	0.15011 (6)	0.79822 (10)	0.0194 (3)	
N2	0.62380 (14)	0.28759 (7)	0.88116 (11)	0.0255 (3)	
C1	0.78640 (15)	0.18306 (7)	0.97396 (12)	0.0183 (3)	
C22	0.65775 (15)	0.03661 (7)	0.95391 (12)	0.0186 (3)	
C2	0.63324 (15)	0.16521 (7)	0.91079 (11)	0.0161 (3)	
C23	0.77233 (16)	0.01718 (8)	1.03430 (14)	0.0259 (3)	
H4	0.799	0.0459	1.0965	0.031*	
C3	0.53140 (15)	0.23112 (7)	0.90468 (12)	0.0179 (3)	
C29	0.73140 (16)	0.12822 (7)	0.73867 (12)	0.0199 (3)	
C34	0.29415 (19)	0.42686 (8)	0.98493 (14)	0.0297 (4)	
H16	0.3257	0.4735	0.993	0.036*	
C38	0.19183 (17)	0.15756 (10)	0.74522 (13)	0.0313 (4)	
H21A	0.1091	0.1818	0.7708	0.047*	
H21B	0.1704	0.1081	0.7401	0.047*	
H21C	0.2065	0.1751	0.6726	0.047*	
C31	0.46259 (15)	0.24680 (7)	1.01067 (11)	0.0184 (3)	
H13A	0.4134	0.2048	1.0336	0.022*	
H13B	0.5417	0.2594	1.0706	0.022*	
C35	0.14653 (19)	0.41193 (9)	0.95249 (14)	0.0299 (4)	
H17	0.0771	0.4482	0.9375	0.036*	
C32	0.35185 (15)	0.30495 (7)	0.99304 (11)	0.0178 (3)	
C4	0.40438 (16)	0.22487 (8)	0.80839 (11)	0.0199 (3)	
C21	0.57050 (15)	0.10158 (7)	0.96548 (12)	0.0170 (3)	
H2A	0.5699	0.1113	1.0446	0.02*	
H2B	0.4673	0.0941	0.9317	0.02*	
C36	0.10069 (17)	0.34392 (9)	0.94210 (12)	0.0252 (3)	
H18	−0.0006	0.3336	0.9211	0.03*	
C28	0.90829 (16)	0.21203 (9)	1.15173 (13)	0.0257 (3)	
H10A	0.962	0.1692	1.1718	0.039*	
H10B	0.8836	0.2345	1.2183	0.039*	
H10C	0.97	0.2429	1.1144	0.039*	
C27	0.62157 (19)	−0.00584 (8)	0.86285 (14)	0.0267 (3)	
H8	0.5444	0.0072	0.8069	0.032*	
C33	0.39630 (17)	0.37363 (8)	1.00568 (12)	0.0227 (3)	
H15	0.497	0.3842	1.0286	0.027*	
C25	0.8090 (2)	−0.08616 (9)	0.93408 (19)	0.0419 (5)	
H6	0.8596	−0.1284	0.9281	0.05*	
C39	0.65003 (16)	0.34293 (8)	0.85183 (12)	0.0202 (3)	
C26	0.6972 (2)	−0.06717 (9)	0.85291 (17)	0.0390 (4)	
H7	0.6718	−0.0959	0.7905	0.047*	
C37	0.20244 (16)	0.29063 (8)	0.96224 (12)	0.0208 (3)	
H19	0.1701	0.2441	0.955	0.025*	
C24	0.84797 (18)	−0.04410 (9)	1.02404 (17)	0.0371 (4)	
H5	0.9265	−0.057	1.079	0.045*	

### Atomic displacement parameters (Å^2^)

**Table:**

	*U*^11^	*U*^22^	*U*^33^	*U*^12^	*U*^13^	*U*^23^
S1	0.0265 (2)	0.0405 (3)	0.0326 (2)	−0.00216 (17)	0.01345 (16)	−0.01572 (18)
S2	0.0424 (2)	0.0188 (2)	0.0414 (2)	−0.00374 (17)	0.01371 (19)	0.00715 (17)
O1	0.0172 (5)	0.0254 (6)	0.0216 (5)	−0.0007 (4)	0.0019 (4)	−0.0027 (4)
O3	0.0183 (5)	0.0277 (6)	0.0195 (5)	0.0007 (4)	0.0004 (4)	0.0027 (4)
O2	0.0178 (5)	0.0301 (6)	0.0311 (6)	−0.0020 (4)	0.0084 (4)	−0.0050 (5)
O4	0.0438 (7)	0.0274 (6)	0.0182 (5)	0.0100 (5)	0.0060 (5)	0.0058 (4)
N1	0.0202 (6)	0.0190 (6)	0.0200 (6)	0.0020 (5)	0.0058 (5)	0.0003 (5)
N2	0.0267 (7)	0.0176 (7)	0.0352 (7)	0.0015 (5)	0.0146 (6)	0.0034 (5)
C1	0.0192 (7)	0.0136 (6)	0.0227 (7)	0.0003 (5)	0.0048 (5)	−0.0003 (5)
C22	0.0165 (6)	0.0144 (7)	0.0267 (7)	0.0000 (5)	0.0094 (5)	0.0032 (5)
C2	0.0170 (6)	0.0147 (6)	0.0175 (6)	0.0011 (5)	0.0058 (5)	−0.0001 (5)
C23	0.0186 (7)	0.0245 (8)	0.0353 (9)	0.0010 (6)	0.0063 (6)	0.0100 (6)
C3	0.0184 (6)	0.0147 (6)	0.0219 (7)	0.0008 (5)	0.0076 (5)	0.0022 (5)
C29	0.0188 (7)	0.0183 (7)	0.0224 (7)	−0.0028 (6)	0.0021 (6)	−0.0016 (5)
C34	0.0368 (9)	0.0180 (7)	0.0361 (9)	0.0048 (7)	0.0119 (7)	0.0001 (6)
C38	0.0202 (7)	0.0512 (11)	0.0207 (7)	0.0038 (7)	−0.0039 (6)	0.0031 (7)
C31	0.0192 (7)	0.0186 (7)	0.0183 (7)	0.0030 (5)	0.0058 (5)	−0.0003 (5)
C35	0.0323 (8)	0.0281 (9)	0.0303 (8)	0.0149 (7)	0.0075 (7)	0.0046 (7)
C32	0.0205 (7)	0.0181 (7)	0.0156 (6)	0.0035 (5)	0.0053 (5)	−0.0005 (5)
C4	0.0240 (7)	0.0207 (7)	0.0165 (7)	0.0075 (6)	0.0083 (5)	0.0011 (5)
C21	0.0151 (6)	0.0139 (6)	0.0227 (7)	−0.0004 (5)	0.0052 (5)	0.0018 (5)
C36	0.0207 (7)	0.0344 (9)	0.0205 (7)	0.0076 (6)	0.0029 (6)	−0.0003 (6)
C28	0.0173 (7)	0.0307 (9)	0.0279 (8)	−0.0013 (6)	−0.0017 (6)	−0.0068 (6)
C27	0.0333 (8)	0.0177 (7)	0.0308 (8)	−0.0034 (6)	0.0105 (7)	−0.0008 (6)
C33	0.0237 (7)	0.0200 (7)	0.0254 (7)	0.0007 (6)	0.0071 (6)	−0.0034 (6)
C25	0.0413 (10)	0.0184 (8)	0.0743 (14)	0.0109 (7)	0.0379 (10)	0.0125 (9)
C39	0.0210 (7)	0.0205 (7)	0.0195 (7)	0.0025 (6)	0.0048 (5)	0.0001 (6)
C26	0.0543 (12)	0.0188 (8)	0.0510 (11)	−0.0039 (8)	0.0326 (10)	−0.0049 (7)
C37	0.0214 (7)	0.0227 (7)	0.0187 (7)	0.0012 (6)	0.0050 (5)	−0.0025 (5)
C24	0.0212 (8)	0.0314 (9)	0.0616 (12)	0.0088 (7)	0.0159 (8)	0.0240 (9)

### Geometric parameters (Å, º)

**Table:**

S1—C29	1.5762 (15)	C38—H21B	0.98
S2—C39	1.5813 (15)	C38—H21C	0.98
O1—C1	1.3320 (17)	C31—C32	1.5132 (19)
O1—C28	1.4531 (17)	C31—H13A	0.99
O3—C4	1.3302 (18)	C31—H13B	0.99
O3—C38	1.4583 (17)	C35—C36	1.385 (2)
O2—C1	1.1998 (17)	C35—H17	0.95
O4—C4	1.1955 (18)	C32—C33	1.396 (2)
N1—C29	1.1818 (19)	C32—C37	1.398 (2)
N1—C2	1.4449 (17)	C21—H2A	0.99
N2—C39	1.168 (2)	C21—H2B	0.99
N2—C3	1.4380 (19)	C36—C37	1.392 (2)
C1—C2	1.5470 (19)	C36—H18	0.95
C22—C23	1.391 (2)	C28—H10A	0.98
C22—C27	1.391 (2)	C28—H10B	0.98
C22—C21	1.5099 (19)	C28—H10C	0.98
C2—C21	1.5528 (19)	C27—C26	1.391 (2)
C2—C3	1.5788 (19)	C27—H8	0.95
C23—C24	1.391 (2)	C33—H15	0.95
C23—H4	0.95	C25—C26	1.378 (3)
C3—C4	1.546 (2)	C25—C24	1.380 (3)
C3—C31	1.5520 (19)	C25—H6	0.95
C34—C35	1.388 (2)	C26—H7	0.95
C34—C33	1.394 (2)	C37—H19	0.95
C34—H16	0.95	C24—H5	0.95
C38—H21A	0.98		
			
C1—O1—C28	117.23 (11)	C36—C35—H17	120.1
C4—O3—C38	117.49 (12)	C34—C35—H17	120.1
C29—N1—C2	145.87 (13)	C33—C32—C37	118.70 (13)
C39—N2—C3	156.08 (15)	C33—C32—C31	121.06 (13)
O2—C1—O1	125.58 (13)	C37—C32—C31	120.23 (13)
O2—C1—C2	124.86 (13)	O4—C4—O3	126.41 (14)
O1—C1—C2	109.54 (11)	O4—C4—C3	124.16 (14)
C23—C22—C27	118.89 (14)	O3—C4—C3	109.32 (11)
C23—C22—C21	121.17 (14)	C22—C21—C2	112.99 (11)
C27—C22—C21	119.92 (13)	C22—C21—H2A	109
N1—C2—C1	107.94 (11)	C2—C21—H2A	109
N1—C2—C21	110.55 (11)	C22—C21—H2B	109
C1—C2—C21	109.01 (11)	C2—C21—H2B	109
N1—C2—C3	105.34 (11)	H2A—C21—H2B	107.8
C1—C2—C3	109.42 (11)	C35—C36—C37	120.26 (15)
C21—C2—C3	114.38 (11)	C35—C36—H18	119.9
C24—C23—C22	120.31 (17)	C37—C36—H18	119.9
C24—C23—H4	119.8	O1—C28—H10A	109.5
C22—C23—H4	119.8	O1—C28—H10B	109.5
N2—C3—C4	107.98 (12)	H10A—C28—H10B	109.5
N2—C3—C31	109.72 (12)	O1—C28—H10C	109.5
C4—C3—C31	107.84 (11)	H10A—C28—H10C	109.5
N2—C3—C2	105.42 (11)	H10B—C28—H10C	109.5
C4—C3—C2	110.55 (11)	C26—C27—C22	120.68 (17)
C31—C3—C2	115.12 (11)	C26—C27—H8	119.7
N1—C29—S1	172.86 (14)	C22—C27—H8	119.7
C35—C34—C33	120.13 (15)	C34—C33—C32	120.57 (15)
C35—C34—H16	119.9	C34—C33—H15	119.7
C33—C34—H16	119.9	C32—C33—H15	119.7
O3—C38—H21A	109.5	C26—C25—C24	120.37 (16)
O3—C38—H21B	109.5	C26—C25—H6	119.8
H21A—C38—H21B	109.5	C24—C25—H6	119.8
O3—C38—H21C	109.5	N2—C39—S2	174.30 (14)
H21A—C38—H21C	109.5	C25—C26—C27	119.70 (18)
H21B—C38—H21C	109.5	C25—C26—H7	120.2
C32—C31—C3	111.59 (11)	C27—C26—H7	120.1
C32—C31—H13A	109.3	C36—C37—C32	120.54 (14)
C3—C31—H13A	109.3	C36—C37—H19	119.7
C32—C31—H13B	109.3	C32—C37—H19	119.7
C3—C31—H13B	109.3	C25—C24—C23	120.04 (17)
H13A—C31—H13B	108	C25—C24—H5	120
C36—C35—C34	119.79 (14)	C23—C24—H5	120
			
C28—O1—C1—O2	−0.6 (2)	C3—C31—C32—C33	−85.99 (16)
C28—O1—C1—C2	177.90 (12)	C3—C31—C32—C37	93.19 (15)
C29—N1—C2—C1	30.4 (3)	C38—O3—C4—O4	−1.7 (2)
C29—N1—C2—C21	−88.8 (3)	C38—O3—C4—C3	174.64 (12)
C29—N1—C2—C3	147.2 (2)	N2—C3—C4—O4	−9.50 (19)
O2—C1—C2—N1	−0.79 (19)	C31—C3—C4—O4	109.00 (16)
O1—C1—C2—N1	−179.25 (11)	C2—C3—C4—O4	−124.35 (15)
O2—C1—C2—C21	119.32 (15)	N2—C3—C4—O3	174.10 (11)
O1—C1—C2—C21	−59.14 (14)	C31—C3—C4—O3	−67.40 (14)
O2—C1—C2—C3	−114.92 (15)	C2—C3—C4—O3	59.25 (14)
O1—C1—C2—C3	66.61 (14)	C23—C22—C21—C2	92.30 (16)
C27—C22—C23—C24	−0.7 (2)	C27—C22—C21—C2	−89.27 (16)
C21—C22—C23—C24	177.70 (13)	N1—C2—C21—C22	52.14 (15)
C39—N2—C3—C4	43.4 (4)	C1—C2—C21—C22	−66.35 (15)
C39—N2—C3—C31	−73.9 (4)	C3—C2—C21—C22	170.82 (11)
C39—N2—C3—C2	161.5 (3)	C34—C35—C36—C37	−1.1 (2)
N1—C2—C3—N2	−73.95 (13)	C23—C22—C27—C26	0.9 (2)
C1—C2—C3—N2	41.85 (14)	C21—C22—C27—C26	−177.57 (14)
C21—C2—C3—N2	164.46 (12)	C35—C34—C33—C32	0.7 (2)
N1—C2—C3—C4	42.50 (14)	C37—C32—C33—C34	−1.8 (2)
C1—C2—C3—C4	158.31 (11)	C31—C32—C33—C34	177.40 (14)
C21—C2—C3—C4	−79.09 (14)	C3—N2—C39—S2	167.5 (12)
N1—C2—C3—C31	165.00 (12)	C24—C25—C26—C27	−1.1 (3)
C1—C2—C3—C31	−79.20 (14)	C22—C27—C26—C25	0.0 (2)
C21—C2—C3—C31	43.41 (16)	C35—C36—C37—C32	−0.1 (2)
C2—N1—C29—S1	18E1 (10)	C33—C32—C37—C36	1.5 (2)
N2—C3—C31—C32	68.64 (15)	C31—C32—C37—C36	−177.71 (13)
C4—C3—C31—C32	−48.75 (15)	C26—C25—C24—C23	1.3 (3)
C2—C3—C31—C32	−172.68 (12)	C22—C23—C24—C25	−0.3 (2)
C33—C34—C35—C36	0.8 (2)		

### Hydrogen-bond geometry (Å, º)

Cg is the centroid of the C32–C37 ring.

**Table:**

*D*—H···*A*	*D*—H	H···*A*	*D*···*A*	*D*—H···*A*
C38—H21*C*···*Cg*^i^	0.98	2.61	3.461 (2)	145

Symmetry code: (i) *x*, −*y*+1/2, *z*+1/2.
